# An Evaluation of the Effect of Dimple Insoles on Foot Temperature in Diabetic Patients

**DOI:** 10.3390/s25051623

**Published:** 2025-03-06

**Authors:** Asma Aferhane, Hassan Douzi, Rachid Harba, Luis Vilcahuaman, Alejandro J. Almenar-Arasanz, Javier Alfaro-Santafé, Hugo Arbañil, María Teresa Arista, Roozbeh Naemi

**Affiliations:** 1 IRF-SIC Laboratory, Ibn Zohr University, Agadir P.O. Box 8106, Morocco; h.douzi@uiz.ac.ma; 2 PRISME Laboratory, Orléans University, 45100 Orléans, France; rachid.harba@univ-orleans.fr; 3 Department of Bioengineering, Pontifical Catholic University of Peru PUCP, Lima 15088, Peru; lvilcah@pucp.edu.pe; 4 Department of Research & Innovation, Podoactiva, Technology Park Walqa, Ctra N 330 a Km 566, Cuarte, 22197 Huesca, Spain; 5 Physiotherapy Department, Campus Universitario, St. Jorge University, Autovía Mudéjar, Km. 299, Villanueva de Gállego, 50830 Zaragoza, Spain; 6 Hospital Nacional Dos de Mayo, Lima 15088, Peru; 7 Centre for Biomechanics and Rehabilitation Technologies, School of Health Science and Wellbeing, Staffordshire University, Leek Road, Stoke-on-Trent ST4 2DF, UK; 8 Centre for Human Movement and Rehabilitation, School of Health and Society, University of Salford, Fredrick Road, Manchester M6 6PU, UK

**Keywords:** diabetic foot, thermal imaging, foot temperature, insoles, walking, non-parametric tests

## Abstract

Objective: Insoles play a crucial role in foot comfort, with their effect on foot temperature being a key factor. This study aims to evaluate and compare the effect of walking with two different insole types—dimple insoles versus a conventional insole—on foot temperature changes in patients with diabetic neuropathy. Methods: Thermal imaging was used to measure the foot temperature of nine participants immediately before and after walking 250 m in each insole. Temperature variations were analyzed for the whole foot across four specific regions to assess and compare the effect of each insole on foot temperature. Results: The Wilcoxon Signed-Rank Test revealed that contralateral temperature differences between the left and right feet after walking ($\left|∆T_{After}\right|$) were significantly (p<0.05) lower in dimple insoles compared to the conventional insoles. This effect was particularly strong in the midfoot and toe regions. Conclusions: The results indicate that insole type can influence foot contralateral temperature differences after walking. These findings provide valuable insights for selecting insoles based on thermal data and can have implications in improving patient outcomes.

## 1. Introduction

Diabetes mellitus (DM), commonly known as diabetes, is one of the fastest-growing chronic diseases worldwide [1]. If not properly managed, this condition can lead to significant complications, including retinopathy, neuropathy, nephropathy, ischemic heart disease, cerebrovascular disorders [2,3], and, in particular, to diabetic foot (DF) disease [2,4]. Diabetic foot ulcers (DFUs) are among the most frequent and serious complications faced by individuals with diabetes mellitus (DM). Patients with DFUs have a mortality risk that is 2.5 times higher than those without these ulcers [5]. Furthermore, at least half of DFUs become infected, often necessitating amputation [6]. The risk of amputations and ulcerations rises with age, leading to severe consequences for patients [6]. DFUs are open wounds that develop due to nerve damage (neuropathy) and reduced blood circulation, which slow down the healing process [7].

On the other hand, foot temperature variations are often associated with the development of foot ulcers, which can be identified using thermal cameras [4]. Thermography is a fast, cost-effective, non-invasive, and contactless technique that is used to visualize the temperature distribution across the plantar surface of the foot [8]. Elevated plantar temperatures have been strongly correlated with diabetic foot complications [9,10]. Notably, Lavery et al. demonstrate the effectiveness of using a 2.2 °C temperature difference between the right and left feet as a standard threshold for detecting potential ulcers [11].

To prevent DFUs, effective interventions must be developed, and prevention is often considered as the best approach. Hence, it is critical to understand the underlying causes of these complications, specifically where high plantar temperature is often identified as a key factor in the development of DFUs [12]. A study conducted by Yavus et al. [13] indicates that plantar temperatures in both healthy individuals and diabetic patients can quickly rise to critical levels after brief weight-bearing exercises, especially due to shear stress on the foot. Furthermore, a recent systematic review revealed that both dermal infrared thermometry and therapeutic footwear with offloading insoles are among the most effective strategies for preventing diabetic foot ulcers [14].

**Figure 1:**
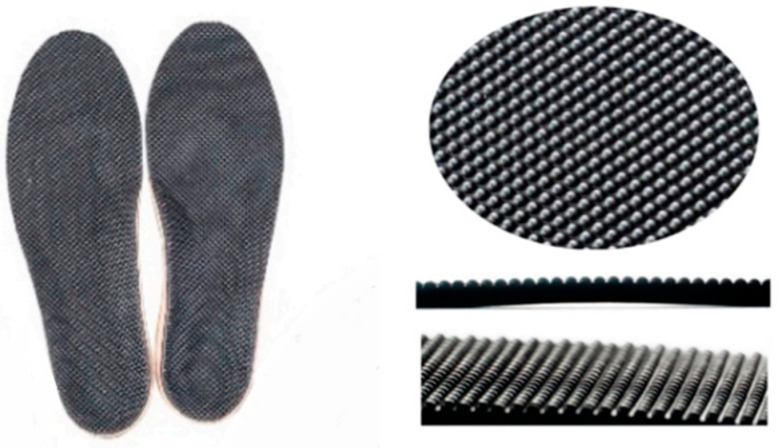
Dimple insoles and close-up of the alignment of dimples.

Insoles have become a primary intervention strategy to reduce plantar pressure and prevent diabetic foot complications by regulating plantar foot temperature [15]. The design of these insoles, particularly their ability to evenly distribute pressure across the foot, is crucial in maintaining foot temperature. More recently, advancements in insole technology aim not only to distribute mechanical stress but also to regulate plantar temperature, providing promising preventive solutions for diabetic foot complications. For instance, a study by Yavuz et al. [16] introduced a novel shoe insole including a cooling unit, a mini-water pump, a battery pack, and a microcontroller designed to both regulate plantar temperatures and reduce pressure concentrations. This insole aimed to maintain skin temperatures within a range shown in animal studies to preserve tissue health. However, this required considerable equipment to be carried by the patient, hence reducing its applicability in real-world settings. Similarly, Perez et al. [17] evaluated the impact of different orthopedic insoles on plantar pressure and temperature distribution during running in healthy individuals, demonstrating that absolute temperatures after running were consistently always greater than before, due to increased mechanical stress on the foot in the presence of inadequate diabetic foot insoles. Hence, there is a need for appropriate insole solutions, one of which could be a dimple insole (Figure 1), to regulate foot temperature during daily life activities.

This study aimed to evaluate the effect of wearing dimple insoles on foot temperature in diabetic patients. To achieve this, a novel approach using contralateral temperature differences after walking, as well as at the assessment of the multitemporal distribution across the whole foot and regions of interest, was utilized. The primary objective was to investigate how walking with dimple insoles affects plantar temperature immediately after walking, in comparison to conventional insoles. Additionally, the study also aimed to investigate the distribution of multitemporal temperature differences to determine whether dimple insoles provide more effective temperature regulation than conventional insoles.

## 2. Materials and Methods

This section presents the methodologies used to evaluate the effect of both dimple and conventional insoles on foot temperature in diabetic patients. It includes the number of participants, the experimental procedures, and the protocols for data collection. Using specific techniques and instruments, thermal images were captured before and after walking with both types of insoles. The thermal data obtained were analyzed to assess changes in foot temperature.

### 2.1. Participants

The inclusion criteria were participants diagnosed with type 2 diabetes and peripheral neuropathy. Individuals with peripheral arterial disease (PAD), a history of lower limb amputation, or active foot ulcers (as exclusion criteria) were excluded from the study. A total of nine participants (*n* = 9, F/M:2/7) were involved in this study, with a mean age of 58±13.81 years (range 30–75 years). Ethical approval for the study was obtained from the Biomedical Research Ethics Committee CEIB of Hospital Nacional HNDM (Evaluation No. 041-2022-CEIB-HNDM, 25 August 2022). All participants received information about the purpose of this study, procedures, and potential risks, and each participant provided written informed consent, demonstrating their voluntary agreement to participate.

### 2.2. Randomization Process

The selection process in the study involved participants pulling a card from a bag to determine whether they would start with the dimple or conventional insole. Afterward, the corresponding insole was placed into the participant’s shoe. A 5 min washout period between the final measurement of the first insole and the baseline measurement of the second insole was used to ensure thermal stability before baseline thermal imaging [18]. During the acclimatization period, participants were asked to remove their shoes and socks and rest in a reclining position for at least 5 min to facilitate thermal stabilization.

### 2.3. Experimental Area and Materials

**Figure 2:**
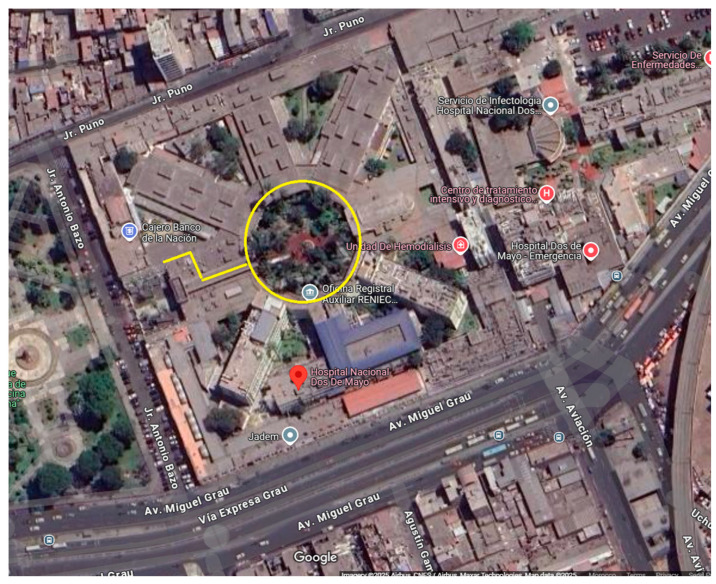
The walking path at the HNDM, Lima, site.

In this study, each participant walked in an outdoor area around the large square at Hospital Nacional Dos De Mayo (HNDM) (as shown in Figure 2). The route was designed to cover the maximum possible distance within this space, ensuring a consistent walking environment. The walkway was carefully selected to provide a uniform and uninterrupted path, allowing participants to complete their walking tasks under consistent conditions.

**Figure 3:**
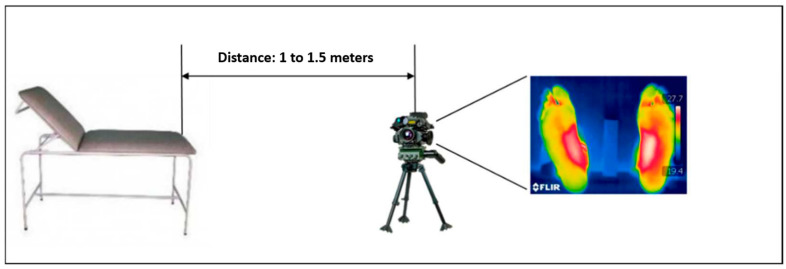
Tools and equipment for thermal imaging acquisition.

A standard medical stretcher was used to comfortably position the participants during thermal imaging (Figure 3). A FLIR ONE Pro thermal imaging camera was employed to capture detailed thermal images of the feet, chosen for accurately detecting temperature variations [19]. The camera was positioned at a distance of 1 to 1.5 m from the feet (Figure 3), with adjustments made according to the size of each foot to ensure the entire foot was within the camera’s field of view, thereby optimizing image quality and ensuring consistency in data collection.

### 2.4. Study Procedure

**Figure 4:**
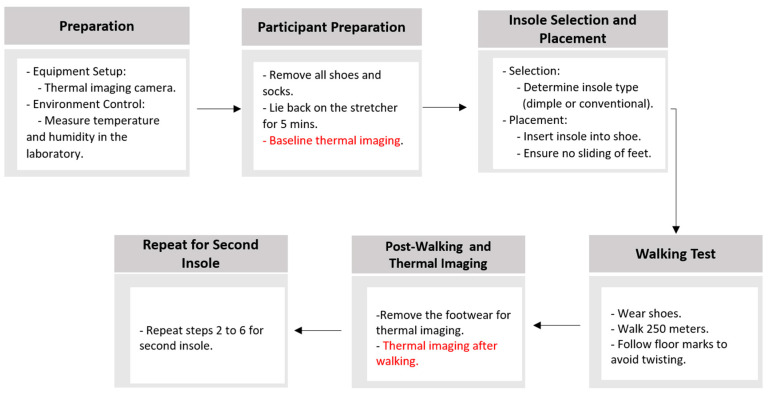
An illustrative outline of the main procedure for foot temperature imaging before and after walking in dimple and conventional insoles.

Two different insole models were used for the experiment: dimple and conventional insoles. The objective of this study was to analyze the temperature elevation of each foot before and after walking and to determine whether a difference between the dimple and conventional insoles can be detected using thermal imaging. To achieve this, the following procedure was used (Figure 4):

The participant was asked to remove their shoes and socks and to lie on the table in a resting position for 5 min for foot acclimatization. Baseline thermal images were captured with the thermal imaging camera. All insoles had a custom insole base produced using a conventional manufacturing technique; they were made of polyamide and created using 3D-HP Multi Jet printing. The dimple insoles have a dimple top cover [20], while the standard insoles have a smooth Poron top cover.

For each test, the patients put on the test shoe and confirmed a good fit. The walking test was performed by having the participant walk at a natural pace for 250 m, following the floor markings and taking care to avoid twisting motions that can cause increased friction inside the shoe. Once the participant had walked 250 m (as a complete round-trip), the participant returned to the couch and removed their footwear. A second thermal image of each participant’s feet was taken immediately after walking. Afterwards, the participant was asked to remain on the couch for 5 min to acclimatize. The entire process was repeated with the second insole to enable a comparative assessment of their effects on foot temperature.

In order to guarantee consistency across measurements, the test was conducted in a controlled clinic room environment (room temperature: 21±1 °C). The temperature and humidity of the room were carefully monitored to minimize any environmental influences on the foot temperature. Prior to measurements, participants were instructed to remove all shoes and socks and rest in a reclining position for at least 5 min to allow for acclimatization [18]. This rest period was adequate to stabilize foot temperature and minimize any effects from previous physical activity or environmental changes. These standardization procedures ensured that the foot temperature readings were not confounded by external factors.

### 2.5. Thermal Imaging and Data Extraction

This subsection outlines the preprocessing of the thermal images acquired with both types of insoles before and after walking, as described in the previous section. It also details the extraction of temperature data from the whole foot and specific foot regions for further analysis.

#### 2.5.1. Preprocessing

**Figure 5:**
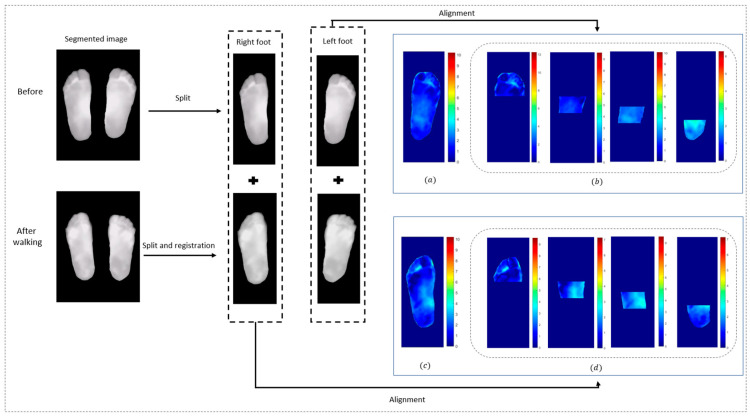
Multitemporal thermal imaging preprocessing. (**a**,**c**) Temperature difference maps for the whole foot before and after walking; (**b**,**d**) temperature difference maps for four specific foot regions.

**Figure 6:**
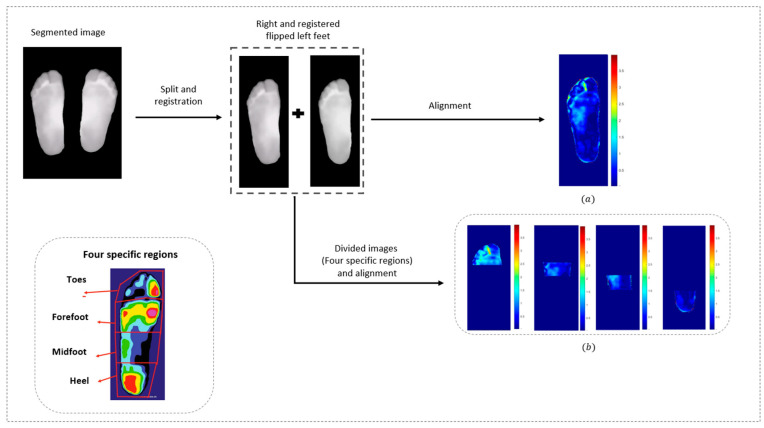
Contralateral thermal imaging preprocessing. (**a**) Temperature difference map for the whole foot between right and left feet; (**b**) Thermal difference maps for four foot regions between left and right feet.

In the context of thermal information extraction, several key steps are required, as detailed in Figure 5 and Figure 6, starting with segmentation. This process is performed using MATLAB 2023a tools, aiming to separate the foot region from the background, subsequently isolating the area of interest. Following segmentation, image registration is a crucial step, ensuring accurate and precise thermal information. In this study, we employed the Affine ConvNet model [21], which has demonstrated significant effectiveness in the registration of thermal images of the plantar foot. The model is based on convolutional neural network (CNN) layers that receive the concatenation of the fixed and moving images to achieve precise alignment.

Once the plantar feet were segmented and registered, the images were divided into four specific regions: the toes, forefoot, midfoot, and heel [22,23]. This division was applied to allow for localized thermal analysis, enabling us to assess temperature variations in different parts of the foot. Following this, temperature maps were generated to visualize these localized thermal effects, providing critical insights for further analysis.

This work consists of two types of analysis—*contralateral analysis*, which compares thermal differences between the left and right foot before and after walking with each insole, where the right foot was used as the fixed image (Figure 6), and *multitemporal analysis*, which examines temperature variations in both feet by comparing images taken before and after a 250 m walk with each insole, with the pre-walking (baseline) images serving as the reference for the registration model (Figure 5). These preprocessing steps yield critical temperature maps, enabling the extraction of thermal data for assessing the effects of dimple and conventional insoles on foot temperature.

#### 2.5.2. Data Extraction

Thermal images were analyzed to measure temperature elevations in the plantar foot. For each participant, temperature data were collected for statistical analysis. In total, four thermal images were obtained for each patient; baseline thermal images were taken before walking for both the dimple and conventional insoles, while the second set of images was taken immediately after walking. Once the preprocessing of images was completed (as detailed in Section 2.3), accurate temperature maps reflecting multitemporal and contralateral temperature differences were generated for each patient (Figure 5 and Figure 6).

These maps are essential for visualizing and quantifying the thermal effects of the insoles on the plantar foot, facilitating the extraction of important thermal information, along with the mean values and standard deviation (SD) of each image.

Contralateral Temperature Differences: For images taken both before and after the walking test for each type of insole, we denoted $\left|∆T_{Before}\right|$ and $\left|∆T_{After}\right|$ to represent the absolute point-to-point temperature differences between the right and left feet at these two instants. These differences were calculated on a pixel-by-pixel basis, resulting in contralateral temperature maps for both images (as shown in Figure 6).

Multitemporal Temperature Differences: The left and right feet are denoted as $\left|∆T_{Left}\right|$ and $\left|∆T_{Right}\right|$ and these represent the absolute point-to-point foot temperature differences between before and after the walking test for the left and right feet, respectively (Figure 5). These differences are calculated for each pixel.

A complete set of thermal information for the whole foot was extracted for each patient using both types of insoles. Table 1 presents the mean temperature values and standard deviation (SD) for each of the four images (baseline and post-walking for each insole), as well as the mean value of the detailed thermal differences (contralateral and multitemporal) with dimple and conventional insoles.

## 3. Study Results and Discussion

This section presents the results of the study, examining the impact of dimple insoles on foot temperature through contralateral distribution and multitemporal analysis. The foot temperature was assessed before and after walking, with statistical analyses performed to determine the significance of the observed changes.

### 3.1. Effect of Insoles

#### 3.1.1. Mean Temperature Analysis

In this study, the effects of two different insoles, dimple and conventional, on foot temperature were analyzed by measuring temperature before and after walking in nine participants. The analysis focused on the variation in mean foot temperature before and after walking for each participant when using the two types of insoles, providing a comparative view of the thermal effects of the dimple and conventional insoles on foot temperature.

**Figure 7:**
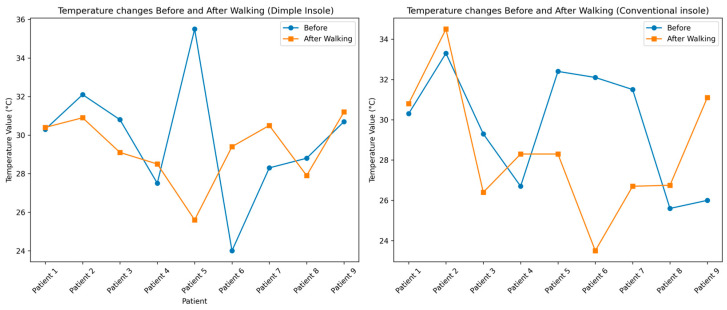
Mean temperature before and after a walking test for dimple and conventional insoles.

Based on Figure 7, the temperature before and after the walking test showed that with the dimple insoles, temperatures changed within a narrower range, from 26 °C to 31 °C, after walking. In contrast, conventional insoles resulted in a broader range of temperature changes, with after-walking temperatures reaching up to 35 °C for participant 2 and dropping to below 24 °C for participant 6. This indicates that the dimple insoles may provide a more consistent thermal response, maintaining foot temperature within a normal variation range compared to conventional insoles. Overall, it appears that the dimple insoles demonstrate a considerable role in thermal regulation during walking.

#### 3.1.2. Contralateral Temperature Differences Distribution

In this section, the impact of two different insoles, dimple and conventional, on foot temperature were examined by analyzing and comparing contralateral thermal data collected from diabetic participants for each insole. The thermal variations, measured as $\left|∆T_{After}\right|$ (temperature difference between feet after walking), provided insights into how each insole affected foot temperature after the walking test.

**Figure 8:**
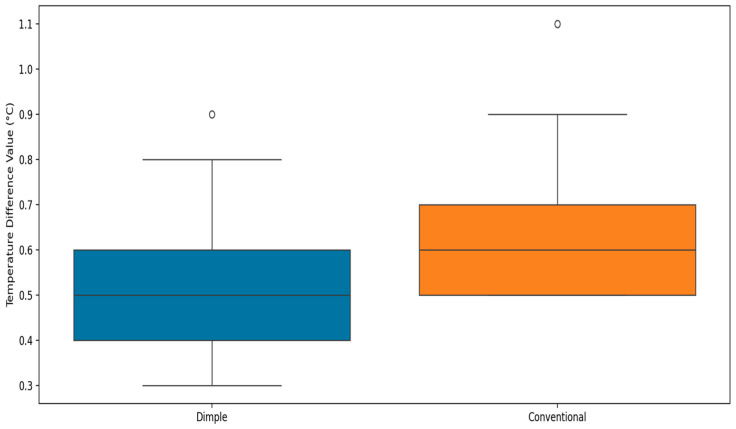
Box plots of contralateral thermal difference variations between right and left feet after walking, for the whole feet with two types of insoles.

As shown in Figure 8, the contralateral temperature differences distribution $\left|∆T_{After}\right|$ between the two insoles can be compared. The Figure 8 provides a clear understanding of how each insole affects foot temperature by comparing the median values in the box plots. The median value for the dimple insole is lower than that for conventional insoles. This suggests that on average, the temperature difference after using the dimple insole is smaller compared to that of conventional insoles. This analysis highlights that while both insoles have similar variability in temperature differences (interquartile range), the dimple insole consistently results in lower temperature difference values compared to the conventional insole. Overall, the dimple insole has a potentially better performance in controlling temperature.

To conduct further analysis, following the contralateral analysis of the overall plantar foot temperature distribution after walking, the authors focus on a more detailed regional analysis, by examining the temperature difference distribution $\left|∆T_{After}\right|$ between the dimple and conventional insoles across four specific regions of the plantar foot, including the toes, forefoot, midfoot, and heel.

**Figure 9:**
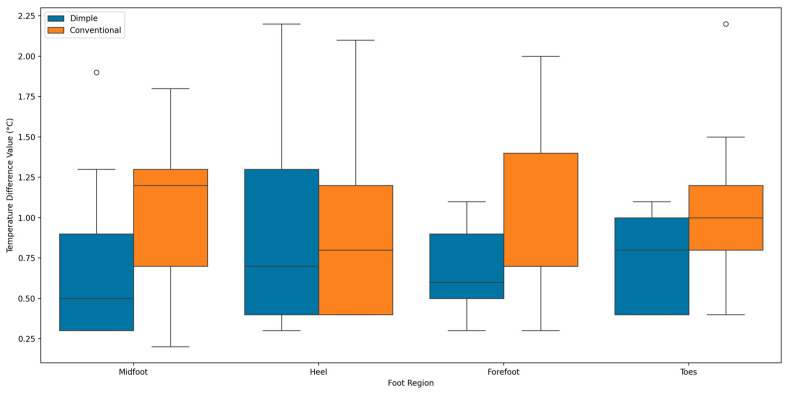
Box plots of contralateral thermal difference variations in four foot regions for right and left feet with dimple and conventional insoles.

As observed in Figure 9, the distribution of $\left|∆T_{After}\right|$ across the whole foot is similar across regions, indicating a lower median temperature difference compared to conventional insoles in these regions, indicating better average temperature control. The overall results indicate that the dimple insoles generally outperform the conventional insoles, with a lower and smaller median temperature difference. This demonstrates the superior capability of dimple insoles in providing even and stable temperature distribution across different foot regions.

#### 3.1.3. Multitemporal Temperature Differences Distribution

In addition to contralateral analysis, the multitemporal temperature differences for left and right feet between the two insoles over time examined. This analysis focused on temperature differences before and after walking, denoted as $\left|∆T_{Left}\right|$ and $\left|∆T_{Right}\right|$ for the left and right feet, respectively, to understand the thermal impact during the walking test.

**Figure 10:**
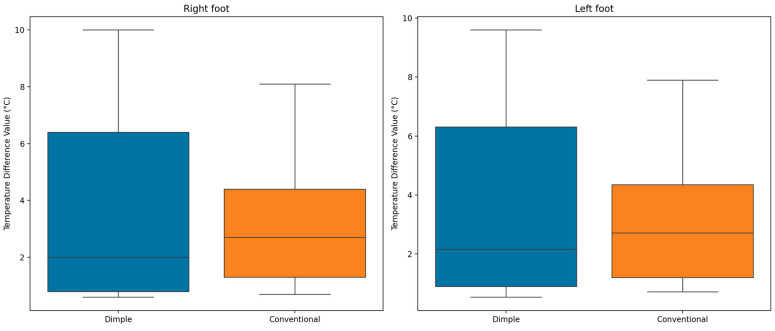
Box plots of multitemporal thermal differences with dimple and conventional insoles for right and left Feet, respectively.

As displayed in Figure 10, the multitemporal temperature difference distribution reveals that dimple insoles consistently resulted in lower median temperature differences compared to conventional insoles, indicating better average temperature control. Despite the lower variability (smaller box) observed for conventional insoles, dimple insoles demonstrate more effective temperature management, as evidenced by their lower median temperature differences. These results further reinforce the findings that dimple insoles may offer a superior performance in maintaining foot temperature stability during walking, as evidenced by its lower multitemporal temperature difference values across multiple foot regions.

### 3.2. Statistical Results

Since the data were not normally distributed, the temperature changes before and after the walking test with each type of insole were analyzed using the Wilcoxon Signed-Rank Test. This non-parametric test was chosen due to the small sample size (*n* = 9) and the non-normal distribution of thermal data. This test was performed on the thermal information of the whole foot and of specific foot regions on both insoles to assess the statistically significant temperature elevation between them based on the extracted information (Table 2).

The results of the Wilcoxon Signed-Rank Test, presented in Table 2, demonstrated that there are no significant differences in multitemporal temperature differences between feet before and after walking, namely $\left|∆T_{Left}\right|$ and $\left|∆T_{Right}\right|$. However, a significant difference was observed between the two insoles (dimple and conventional) in the contralateral temperature of left and right feet after walking in relation to the whole foot and some interesting regions like the toes and midfoot, which are denoted as $\left|∆T_{Before}\right|$ and $\left|∆T_{After}\right|$ with p<0.05.

This finding suggests that contralateral differences after walking $\left|∆T_{After}\right|$ which are a crucial factor in foot health can be affected by using the conventional and dimple insoles. The statistical significance highlights the fact that the choice of insole can markedly influence contralateral temperature changes after walking and the thermal assessment tool and method can distinguish between the dimple and conventional insoles.

### 3.3. Discussion

The dimple insole was designed to provide mechanical stimulation to the tissues of the foot’s sole, promoting improved skin perfusion, as previously established by Behforootan et al. [24]. This exploratory study has demonstrated the insole’s ability to reduce temperature differences between the left and right feet, a key marker for diabetic foot ulceration. In this context, our findings suggest that compared to conventional insoles, the dimple insole may help lower the risk of foot complications.

The results revealed significant contralateral temperature differences after walking $\left|∆T_{After}\right|$ in relation to the whole foot and some specific regions such as the toes and forefoot, which can indicate that the dimple insoles contribute to better circulation and tissue health in these areas. Moreover, a more uniform temperature distribution was observed after wearing these insoles while walking (Section 3.1.1), further suggesting their potential to prevent conditions like diabetic foot ulcers, which are often associated with temperature imbalances. In the forefoot, a significant difference was observed before walking. This effect decreased after walking, as the *p*-value was no longer significant. This indicates that the forefoot’s response to the insole may be further influenced by factors such as shear forces exerted during walking, which could temporarily mask or reduce the temperature changes. The forefoot is subject to high mechanical stress and pressure during movement, likely affecting its thermoregulatory response differently to that of the toes or midfoot.

In contrast, the heel region showed no significant temperature changes before or after walking. This may be due to the anatomical characteristics of the heel, such as the insulating properties of the fat pad, which reduce thermal responsiveness. Additionally, the heel bears less dynamic weight and experiences less shear force during walking compared to other regions, further limiting its responsiveness to insole-induced thermal changes.

On the other hand, although multitemporal temperature differences were not statistically significant, the dimple insoles consistently showed lower median temperature differences compared to the conventional insoles, indicating better temperature control (Section 3.1.3). While the conventional insole had less variability, the dimple insoles proved more effective at stabilizing foot temperature. These findings may suggest that the dimple insoles offer superior temperature regulation, and in that context could potentially decrease the risk of complications like diabetic foot ulcers.

These preliminary results are promising and provide new insights into the role of insole design in relation to improving foot thermal regulation, supporting previous research. Earlier studies emphasized the importance of design elements, such as the presence of air at the interface between the skin and the insole [25]. Both material conductivity and thermal capacity were found to influence foot temperature [25]. The results of this study, showing the dimple insoles’ superior performance compared to conventional designs, confirm that variations in material composition and trapped air in the insole can significantly affect temperature regulation [25]. Moreover, evaluated the effects of dimpled insoles on plantar temperature and pressure distribution [20]. Their objective was to assess how these insoles influenced plantar temperature both during and immediately after walking, with a particular focus on their impact on plantar pressure [20]. The study demonstrated that dimple insoles effectively reduced plantar temperature in diabetic participants compared to healthy individuals [20]. Overall, the findings highlight the potential of dimpled insoles to enhance foot thermal symmetry between the left and right feet, has the potential to lower the risk of diabetic foot ulcers, offering valuable insights into the development of therapeutic insoles designed to prevent foot complications. However, some limitations must be acknowledged. The study was conducted with a relatively small number of participants and focused on diabetic patients with neuropathy. Future research should investigate the long-term impact of insole design on plantar temperature, pressure and balance, particularly in individuals with more advanced foot complications, such as history of diabetic ulceration and peripheral arterial disease.

In the case of the dimple insoles, the observed reduction in temperature, in addition to potentially improved perfusion, can be attributed to the insoles’ ability to better regulate and dissipate heat. Enhanced perfusion may lead to more efficient thermoregulation, preventing excessive heat buildup and thereby reducing surface temperature [26]. Additionally, the dimple insoles’ design could contribute to improved pressure distribution and comfort, further supporting effective temperature control.

Therefore, while improved perfusion typically correlates with higher temperatures in some contexts, the dimple insoles likely promote a more stable and controlled thermal environment, which helps explain the reduced temperature despite enhanced blood flow.

## 4. Conclusions

The dimple insoles were signifcantly more effective in reducing contralateral temperature differences after walking compared to conventional insoles.

In addition, dimple insoles (compared to conventional insoles) demonstrated a consistently more effective temperature control, as evidenced by the lower medianmultitemporal temperature differences. These findings indicates that the dimple insole fulfils the expected ability to better regulate temperature control, that may be due to the ability to stimulate plantar soft tissues and improve skin perfusion during walking. The findings can have potential implications in improving diabetic foot outcomes through appropraite insole design.

## Figures and Tables

**Table 1:** Mean values and standard deviations of whole-foot thermal information with the dimple and conventional insoles.

Thermal Data	Dimple Insoles (°C)	Conventional Insoles (°C)
Temperature before walking $T_{Before}$	29.77 (±3.20)	29.68 (±2.94)
Temperature after walking $T_{After}$	29.77 (±1.77)	28.48 (±3.23)
Temperature difference between feet before walking $\left|∆T_{Before}\right|$	0.61 (±0.27)	0.75 (±0.38)
Temperature difference between feet after walking $\left|∆T_{After}\right|$	0.67 (±0.30)	0.81 (±0.39)
Temperature difference (right foot) $\left|∆T_{Right}\right|$	3.68 (±3.84)	3.17 (±2.41)
Temperature difference (left foot) $\left|∆T_{Left}\right|$	3.68 (±3.78)	3.08 (±2.37)

**Table 2:** Wilcoxon Signed-Rank Test results for thermal data comparing dimple and conventional insoles.

		*p*-Value (Wilcoxon Signed-Rank Test)			
		$\left|∆T_{Before}\right|$	$\left|∆T_{After}\right|$	$\left|∆T_{Right}\right|$	$\left|∆T_{Left}\right|$
	Whole foot	0.058	**0.026**	0.674	0.82
Foot Region	Toes	0.106	**0.041**	0.57	0.73
	Forefoot	**0.017**	0.164	0.67	0.57
	Midfoot	0.39	**0.0356**	0.73	0.91
	Heel	0.482	0.778	0.99	0.99

## Data Availability

Dataset available on request from the authors.
